# A blood cyst in the aortic root after acute myocardial infarction

**DOI:** 10.1093/ehjimp/qyae107

**Published:** 2024-10-23

**Authors:** Masaya Hirayama, Satoshi Kainuma, Takeshi Kitai, Kisaki Amemiya, Satsuki Fukushima

**Affiliations:** Department of Cardiovascular Surgery, National Cerebral and Cardiovascular Center, 6-1 Kishibe-Shinmachi, Suita, Osaka 564-8565, Japan; Department of Cardiovascular Surgery, National Cerebral and Cardiovascular Center, 6-1 Kishibe-Shinmachi, Suita, Osaka 564-8565, Japan; Cardiovascular Medicine, National Cerebral and Cardiovascular Center, Osaka, Japan; Department of Pathology, National Cerebral and Cardiovascular Center, Osaka, Japan; Department of Cardiovascular Surgery, National Cerebral and Cardiovascular Center, 6-1 Kishibe-Shinmachi, Suita, Osaka 564-8565, Japan

**Keywords:** cariogenic shock, acute myocardial infarction, Blood cyst, Thrombus, Impella

A 57-year-old man, who had been supported by venoarterial extracorporeal membrane oxygenation and intra-aortic balloon pumping for 10 days for refractory cardiogenic shock following left main trunk acute myocardial infarction, was referred to our hospital. On admission, transthoracic echocardiography showed severely impaired left ventricular systolic function, while chest radiography revealed pulmonary congestion. Right heart catheterization showed a mean pulmonary artery pressure of 40 mmHg, indicating pulmonary hypertension secondary to left ventricular dysfunction. An upgrade to IMPELLA 5.5 was planned, but preoperative transoesophageal echocardiography unexpectedly detected a mobile, thin-walled, monolocular, echo-free structure without an obvious blood flow signal, resembling a cyst above the noncoronary cusp (*Panel A* and Supplementary data online, *Video S1*). As the possibility of a thrombus could not be ruled out, extraction of the mass followed by IMPELLA 5.5 implantation via the right subclavian artery was scheduled. After achieving cardioplegic arrest, an oblique incision at the aortic root was made. Intraoperative microscopic finding confirmed a thrombus at the noncoronary cusp that was extracted with conservation of the aortic valve (*Panel B* and Supplementary data online, *Video S2*). IMPELLA 5.5 implantation following thrombectomy was performed without any thromboembolic complications. Histological examination revealed that the mass consisted of red blood cells with degeneration, suggesting a blood cyst (*Panel C*). Blood cyst is believed to be the result of microscopic atrial and ventricular endocardial invagination into the atrioventricular and semilunar valves, respectively. Rarely, blood cysts are reported in adults with devastating embolic consequences (e.g. stroke). By reporting this uncommon presentation of a blood cyst—like lesion mimicking a thrombus in the aortic root, we hope clinicians will be better equipped to establish the diagnosis and respond appropriately, as this could have contraindicated IMPELLA insertion and led to thromboembolic events.

**Figure:**
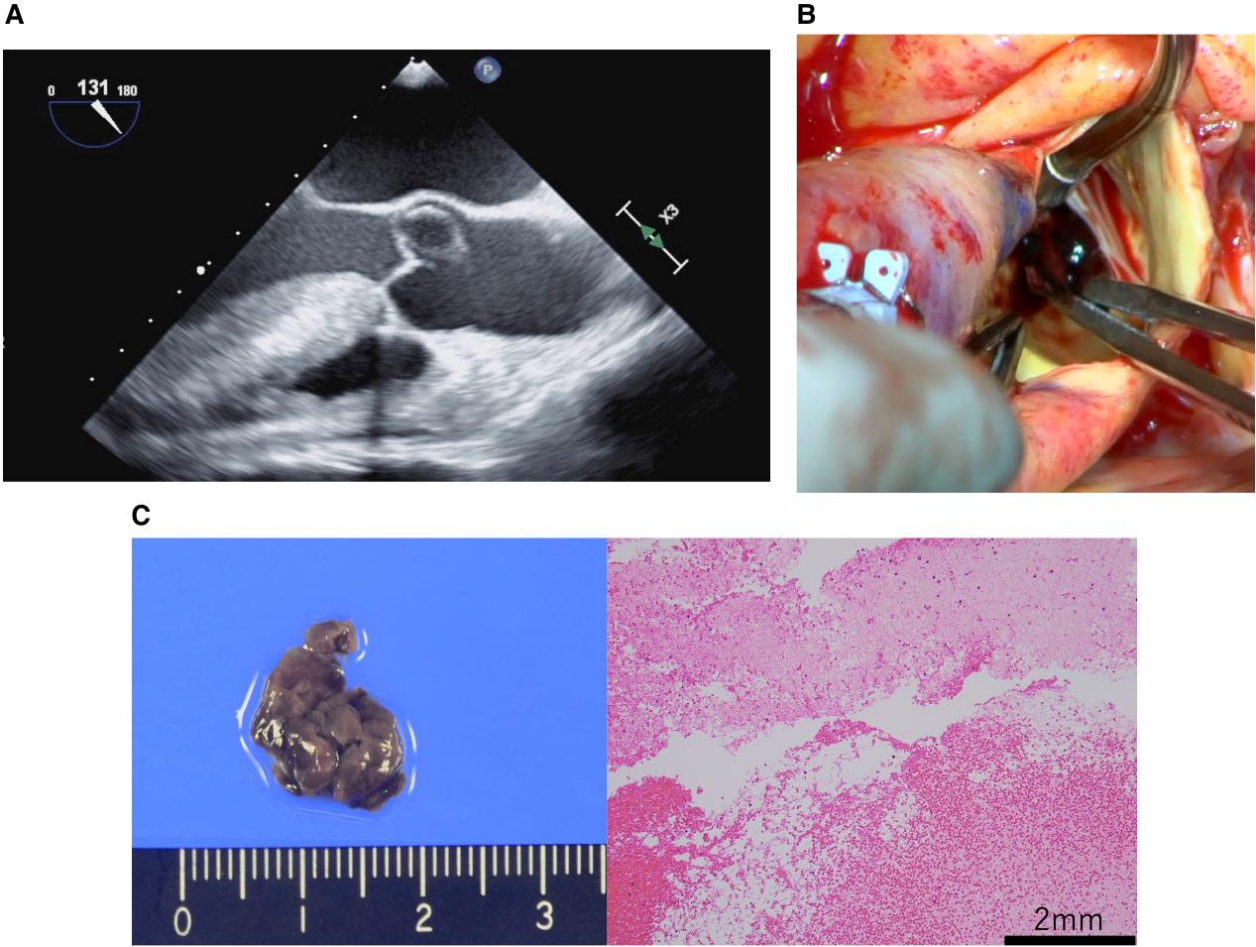


## Supplementary data

Supplementary data are available at *European Heart Journal - Imaging Methods and Practice* online.

**Consent:** The informed consent was given by the patient.

**Funding:** The authors did not receive support from any organization for the submitted work.

**Data availability:** The data underlying this article will be shared on reasonable request to the corresponding author.

## Lead author biography


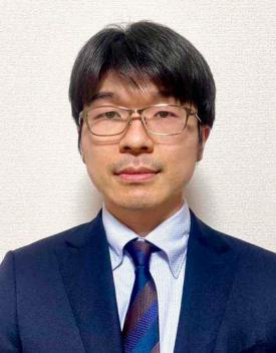


Masaya Hirayama, M.D is a physician in the Department of Cardiac Surgery at National Cerebral and Cardiovascular Center, Japan. He graduated from Kagawa University. He finished cardiovascular surgery residency programme at Kurashiki Central Hospital, and Cardiovascular Surgery Fellowship at National Cerebral and Cardiovascular Center. His current specialty is general adult cardiac surgery, including coronary artery disease, heart valve disease, and heart failure.

## Supplementary Material

qyae107_Supplementary_Data

